# Characterization of Temperate LPS-Binding Bordetella avium Phages That Lack Superinfection Immunity

**DOI:** 10.1128/spectrum.03702-22

**Published:** 2023-05-01

**Authors:** Dorothee Serian, Yury Churin, Jens André Hammerl, Manfred Rohde, Arne Jung, Anja Müller, Min Yue, Corinna Kehrenberg

**Affiliations:** a Institute for Veterinary Food Science, Justus Liebig University Giessen, Giessen, Germany; b Department Biological Safety, German Federal Institute for Risk Assessment, Berlin, Germany; c Central Facility for Microscopy, Helmholtz Centre for Infection Research GmbH, Braunschweig, Germany; d Clinic for Poultry, University of Veterinary Medicine Hannover Foundation, Hannover, Germany; e Institute of Preventive Veterinary Science and Department of Veterinary Medicine, Zhejiang University College of Animal Sciences, Hangzhou, China; f Hainan Institute of Zhejiang University, Sanya, China; University of Tennessee Knoxville

**Keywords:** *Bordetella avium*, bacteriophages, life cycle, phage receptor

## Abstract

Bordetella avium causes a highly infectious upper respiratory tract disease in turkeys and other poultry with high economic losses. Considering the antimicrobial resistance crisis, bacteriophages (phages) may be an alternative approach for treating bacterial infections such as bordetellosis. Here, we describe seven *B. avium* phages, isolated from drinking water and feces from chicken and turkey farms. They showed strong bacteriolytic activity with a broad host range and used lipopolysaccharides (LPS) as a host receptor for their adsorption. All phages are myoviruses based on their structure observed by transmission electron microscopy. Genome sequence analyses revealed genome assembly sizes ranging from 39,087 to 43,144 bp. Their permutated genomes were organized colinearly, with a conserved module order, and were packed according to a predicted headful packing strategy. Notably, they contained genes encoding putative markers of lysogeny, indicative of temperate phages, despite their lytic phenotype. Further investigation revealed that the phages could indeed undergo a lysogenic life cycle with varying frequency. However, the lysogenic bacteria were still susceptible to superinfection with the same phages. This lack of stable superinfection immunity after lysogenization appears to be a characteristic feature of *B. avium* phages, which is favorable in terms of a potential therapeutic use of phages for the treatment of avian bordetellosis.

**IMPORTANCE** To maintain the effectiveness of antibiotics over the long term, alternatives to treat infectious diseases are urgently needed. Therefore, phages have recently come back into focus as they can specifically infect and lyse bacteria and are naturally occurring. However, there is little information on phages that can infect pathogenic bacteria from animals, such as the causative agent of bordetellosis of poultry, *B. avium*. Therefore, in this study, *B. avium* phages were isolated and comprehensively characterized, including whole-genome analysis. Although phenotypically the phages were thought to undergo a lytic cycle, we demonstrated that they undergo a lysogenic phase, but that infection does not confer stable host superinfection immunity. These findings provide important information that could be relevant for potential biocontrol of avian bordetellosis by using phage therapy.

## INTRODUCTION

The Gram-negative, aerobic bacterium Bordetella avium causes a highly infectious upper respiratory tract disease, called bordetellosis, in turkeys and other poultry of all ages (1–4). The pathogen possesses some virulence factors, such as tracheal cytotoxin, dermonecrotic toxin, fimbriae, filamentous hemagglutinin (FHA), and lipopolysaccharide (LPS), which are similar to the virulence factors of other medically important *Bordetella* species. Infections with *B. avium* in poultry include colonization of the trachea and destruction of ciliated tracheal epithelial cells (3, 5, 6), leading to symptoms such as sneezing, coughing, nasal discharge, and swollen infraorbital sinuses (1, 3, 7–9). Diseased animals show reduced body weight gain due to their poor general condition and a higher susceptibility to various secondary infections (3). This leads to high economic losses in commercial poultry farms (3, 6, 10), although the disease is characterized by a high morbidity but low mortality (11, 12). However, infections are usually treated with antimicrobial agents and, thus, resistant and multidrug-resistant *B. avium* isolates have recently been reported (13), demanding new treatment methods.

Given fears of a postantibiotic era, special attention is paid to the most widespread organisms on earth, bacteriophages (phages) (14, 15). As viruses that specifically infect and lyse bacteria, they occur naturally and have shown promising potential in the biocontrol of pathogenic bacteria (16–18) and as a detection tool of foodborne pathogens (19, 20). Many of their properties, such as their high specificity toward target bacteria, their ability to destroy biofilms by matrix rupture, replication in pathogenic bacteria and the fact that they do not interfere with the intended bacterial flora make phages attractive as therapeutic agents. In particular, the treatment of multidrug-resistant (MDR) bacteria with phages is a field that is receiving increasing attention in human medicine (21–23). At the beginning of phage infection of a bacterial cell, the so-called receptor-binding proteins at the distal end of the phage bind to ligands (receptors) on the bacterial surface (24). Structures such as proteins, polysaccharides, LPS, and carbohydrate components, as well as pili and flagella, serve as phage receptors (25–27), with LPS playing an important role in Gram-negative bacteria (25). For phage therapy, particularly, obligatory lytic phages are being used (28, 29). However, temperate phages can undergo both lytic and lysogenic cycles, and after integration of their genomes into bacterial chromosomes, they can substantially influence the phenotypes of host bacteria (30). Through specific gene expression-like repressor molecules or proteins which prevent the injection of further phage DNA into the cell, the prophage often confers increased fitness to the host (31–33).

In *B. avium*, only a temperate transducing prophage Ba1 has been characterized so far, which was assigned to the *Myoviridae* family (34, 35). This study therefore aimed to isolate and characterize *B. avium* phages from the environment of poultry farms. Transmission electron microscopy and genome sequence analyses of the phages were performed, as well as determinations of the host ranges and analysis of their bacteriolytic activity. This characterization was intended to provide information on the possibility of using phages to treat *B. avium* infections.

## RESULTS

### Phage isolation and phenotypic characterization.

**FIG 1:**
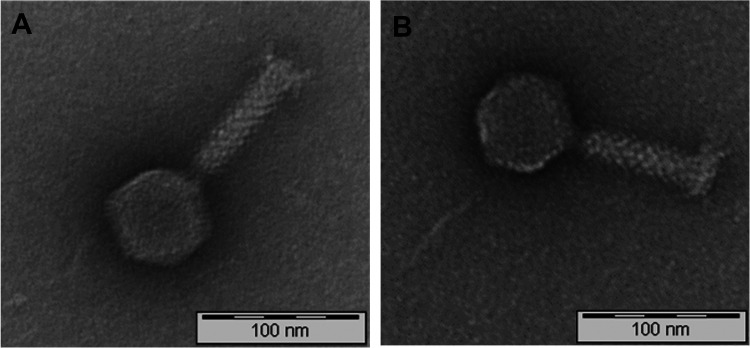
Transmission electron microscopy analysis of phages vB_BaM-IFTN2 (IFTN2) (A) and vB_BaM-IFTN7 (IFTN7) (B).

Seven phages were isolated from drinking water (*n* = 6) and fecal samples (*n* = 1) and purified using the double agar overlay plaque assay method (Table 1). After overnight incubation, phages formed clear plaques with a diameter ranging from 0.1 to 3 mm, depending on strain and phage (data not shown). Phages were designated vB_BaM-IFTN1 to vB_BaM-IFTN7 (abbreviated as IFTN1 to IFTN7) according to the recommendations on phage nomenclature (36, 37). Transmission electron microscopy (TEM) was used to determine phage morphology. All phages showed an icosahedral head (head sizes measured parallel and perpendicular to the tail were ranging from 61 to 57 nm and from 73 to 75 nm, respectively) and a contractile tail part (75 nm to 109 nm in length) (Table 1). TEM pictures of the *B. avium* phages IFTN2 and IFTN7 are shown in Fig. 1. Based on these structural features, which are consistent with the A1 morphotype, the phages have been classified as myoviruses, according to the International Committee on Taxonomy of Viruses (38–40).

**TABLE 1:** Source and characteristics of isolated phages

Phages	Short name	Taxonomy	Host strain	Source of isolation	Head size (nm)	Tail length (nm)^*a*^	Genome assembly sizes (bp)^*b*^
vB_BaM-IFTN1	IFTN1	Myovirus	CCUG 13726^T^	Drinking water, chicken, noncommercial farm	68 × 66	82	39,795
vB_BaM-IFTN2	IFTN2	Myovirus	CCUG 13726^T^	Fecal sample, chicken, noncommercial farm	61 × 57	89	39,087
vB_BaM-IFTN3	IFTN3	Myovirus	12/574/1/C	Drinking water, chicken, noncommercial farm	66 × 69	ND	39,977
vB_BaM-IFTN4	IFTN4	Myovirus	CCUG 13726^T^	Drinking water, chicken, commercial farm	66 × 67	109	42,492
vB_BaM-IFTN5	IFTN5	Myovirus	CCUG 13726^T^	Drinking water, chicken, commercial farm	69 × 69	62	43,144
vB_BaM-IFTN6	IFTN6	Myovirus	16/29/1/B	Drinking water, chicken, noncommercial farm	65 × 71	75	40,308
vB_BaM-IFTN7	IFTN7	Myovirus	X1131/2a	Drinking water, chicken, noncommercial farm	68 × 64	87	40,972

a ND, not determined.

b Genome size of Bordetella avium 197N: 3,732,255 bp; G+C content of 61.58%.

**FIG 2:**
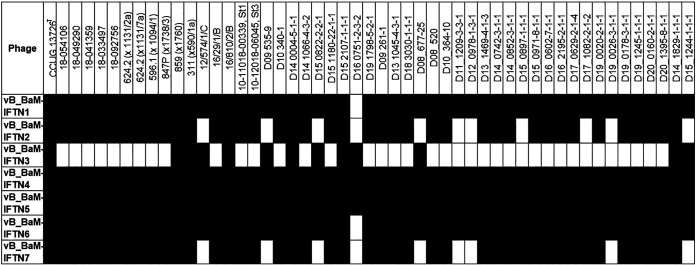
Host range determination. The collection of 51 *B. avium* isolates was evaluated for susceptibility to phages vB_BaM-IFTN1 to vB_BaM-IFTN7 (IFTN 1 to 7). Black bars indicate lysis and white bars indicate no lysis.

The host range of the phages was determined using the spot test on a double agar overlay and the collection of 50 *B. avium* isolates and the *B. avium* type strain CCUG 13726^T^. Spotting high phage titers (about 3 × 10^9^ to 3 × 10^10^ PFU mL^−1^) resulted in a continuous lysed area. After serial dilutions of the phage suspensions, individual clear plaques appeared, as shown for phages IFTN3 and IFTN4 (see Fig. S1 in the supplemental material). An individual clear plaque morphology in the spot assay was defined as positive lysis activity (Fig. 2, black bars). Each *B. avium* isolate was lysed by at least one phage, in most cases even by several phages (Fig. 2). Overall, this assay demonstrated a broad host range of isolated phages.

**FIG 3:**
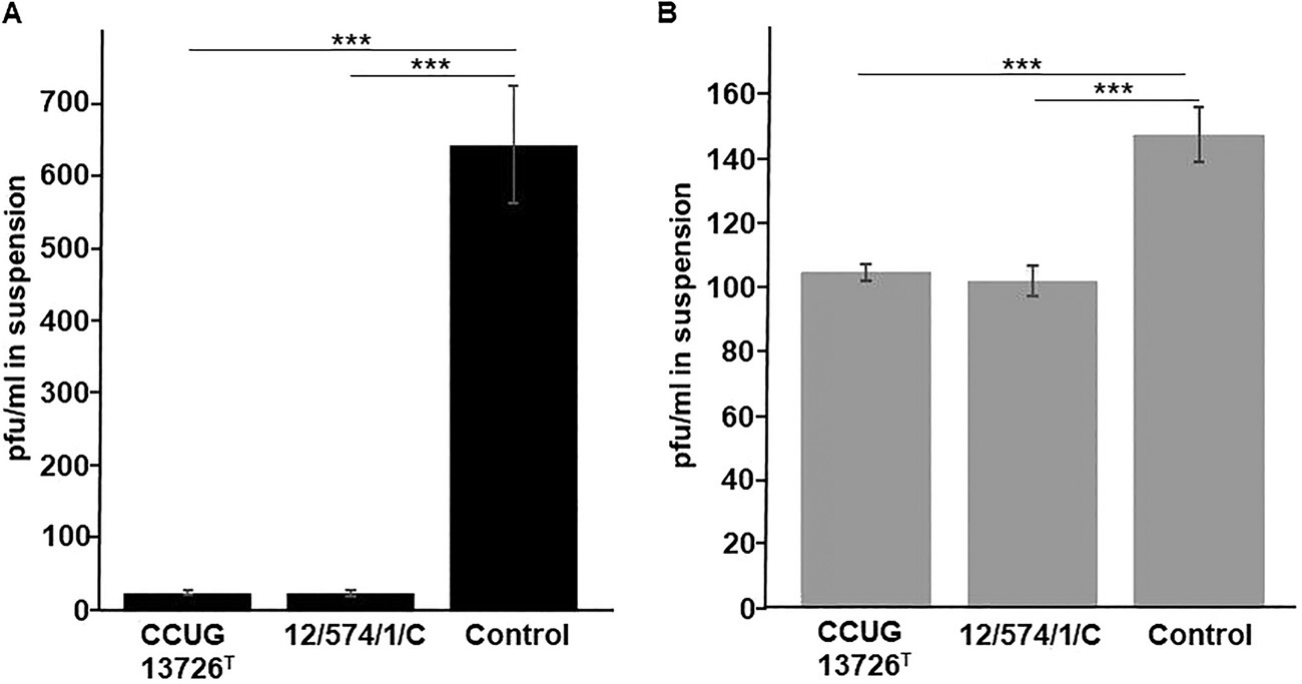
Phage adsorption assay. Phage vB_BaM-IFTN4 (IFTN4) (A) and vB_BaM-IFTN3 (IFTN3) (B) adherence to *B. avium* type strain CCUG 13726^T^ and *B. avium* isolate 12/574/1/C are shown (mean ± SD). ***, *P* < 0.0005. Control samples were included without preincubation with bacteria. The results represent the average of three independent experiments.

**FIG 4:**
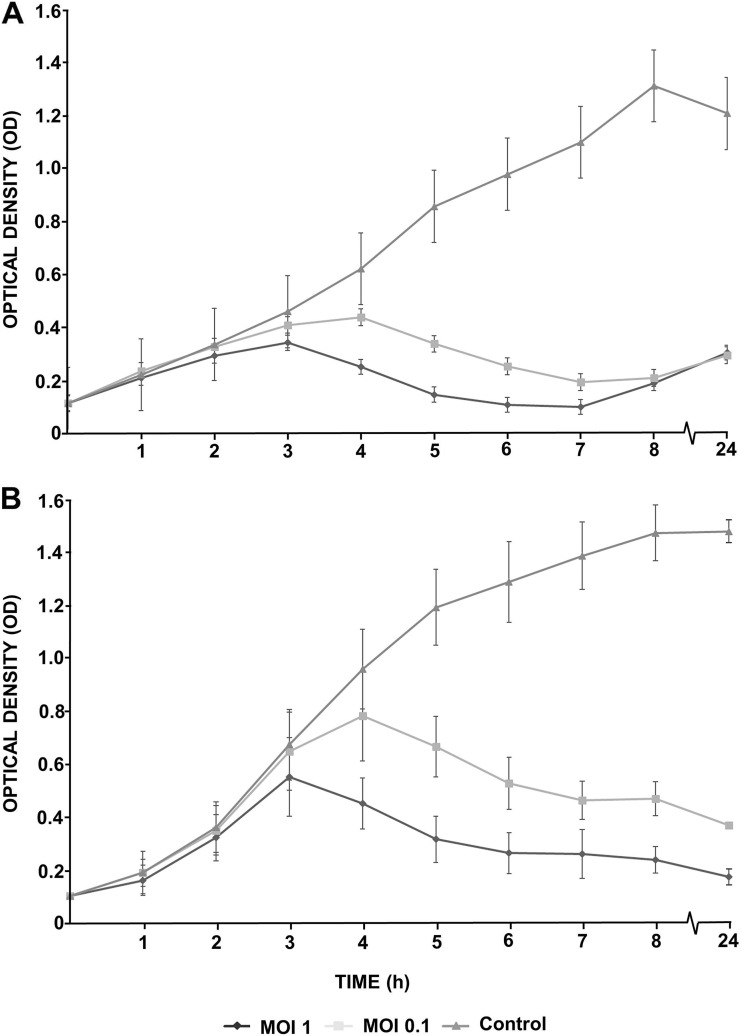
Bacteriolytic activity. The bacteriolytic activity of phage vB_BaM-IFTN4 (IFTN4) to *B. avium* type strain CCUG 13726^T^ (A) and phage vB_BaM-IFTN3 (IFTN3) to *B. avium* isolate 12/574/1/C (B) are shown at MOI 1 and MOI 0.1, in reference to the bacterial growth curve. These data are the average of three independent experiments (mean ± SD).

Characteristics of a phage-host relationship such as phage adherence to the host cell and bacteriolytic activity are basic biological properties of any virus. These features were investigated for phages IFTN3 and IFTN4 using the phage adsorption assay with *B. avium* field isolate 12/574/1/C and *B. avium* type strain CCUG 13726^T^ (Fig. 3). The selection of phages was done based on differences in their host strains (Table 1) and host range (Fig. 2). Both phages, IFTN4 (Fig. 3A) and IFTN3 (Fig. 3B), demonstrated equal adherence to both *B. avium* isolates. To obtain additional information on the phage effectiveness, the bacteriolytic activity was investigated. Fig. 4 and Fig. S2 show the bacteriolytic activity of phage IFTN4 against *B. avium* type strain CCUG 13726^T^ (Fig. 4A, Fig. S2A) and phage IFTN3 against *B. avium* isolate 12/574/1/C (Fig. 4B; Fig. S2B) at multiplicity of infection (MOI) 1 and MOI 0.1, in relation to the bacterial growth curve. Both experiments showed a steadily increasing optical density (OD) and CFU mL^−1^ value of the control sample up to hour 8. However, if phages were added to the bacterial cells at a concentration of MOI 1, lysis of the bacterial cells was observed after 3 h by OD value measurements (Fig. 4A and B) and after 1 h by bacterial colony count determinations. After 3 h, a reduction in bacterial counts by 4 log_10_ steps compared to the control sample was observed (Fig. S2A and B). With an MOI of 0.1, the same effect was observed after 4 h (Fig. 4A and B; Fig. S2A and B). The dependence of the phages on their MOI and the time at which the effect occurred were the same for both phages. However, after infection of *B. avium* type strain CCUG 13726^T^ with phage IFTN4, the OD value started to increase again from hour 7. This phenomenon was observed for both phages after hour 8 when colony counts were determined (Fig. S2A and B). There was a steady increase in colony counts until the last measurement time point at hour 48, indicative for phage-resistant bacterial clones.

### Identification of the phage host receptor.

**FIG 5:**
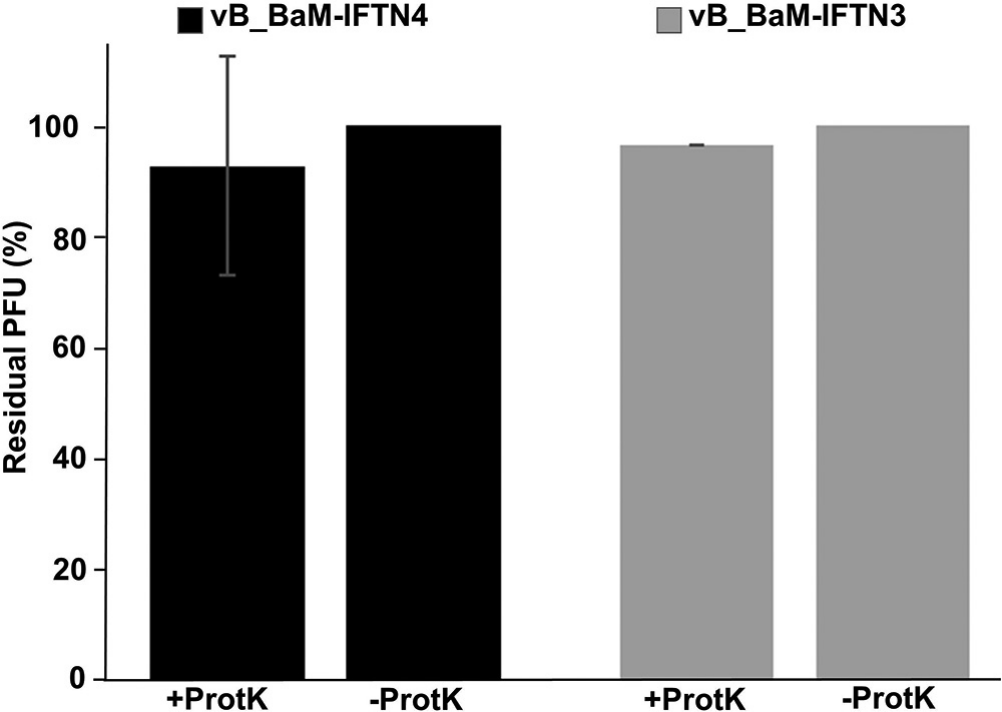
Effect of proteinase K treatment. *B. avium* cells were treated with or without Proteinase K and the adsorption of phage vB_BaM-IFTN4 (IFTN4) to *B. avium* type strain CCUG 13726^T^ (black bars) and phage vB_BaM-IFTN3 (IFTN3) to *B. avium* isolate 12/574/1/C (gray bars) were measured. +ProtK: treatment with proteinase K. –ProtK: control without treatment. Scale bar is shown as residual PFU percentages. These are data of three independent experiments in triplicate for a total of nine repeats per group (mean ± SD).

**FIG 6:**
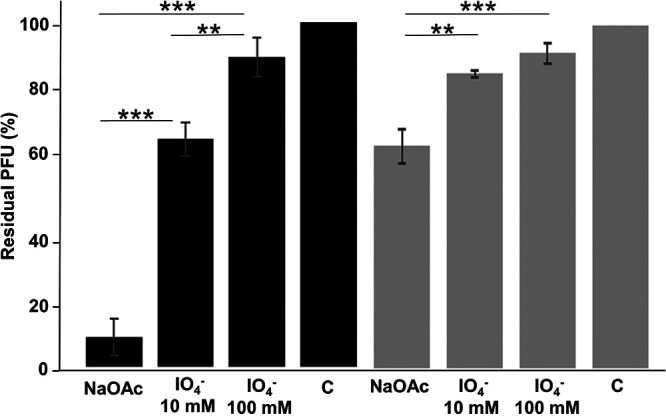
Effect of sodium periodate treatment. *B. avium* cells were treated with different concentrations of sodium periodate and the adsorption of phage vB_BaM-IFTN4 (IFTN4) to *B. avium* type strain CCUG 13726^T^ (black bars) and phage vB_BaM-IFTN3 (IFTN3) to *B. avium* isolate 12/574/1/C (gray bars). C: control without treatment. NaOAc: treatment with sodium acetate (50 mM). IO_4_– 10 mM: treatment with 10 mM sodium periodate. IO_4_– 100 mM: treatment with 100 mM sodium periodate. The scale bar is shown as residual PFU percentages. *, *P* < 0.05; **, *P* < 0.025; ***, *P* < 0.0005. These are data of three independent experiments in triplicate for a total of nine repeats per group (mean ± SD).

To determine whether cell surface proteins or lipopolysaccharides serve as receptor for *B. avium* phages, bacterial treatment with proteinase K (degradation of proteins) or sodium periodate (oxidation of polysaccharides) was performed, followed by a phage adsorption assay. Treatment of bacterial cells with proteinase K did not lead to a significant change in phage adherence (Fig. 5). After proteinase K treatment, the residual PFU were still 92% for phage IFTN4 (Fig. 5, black bars) and 95% for phage IFTN3 (Fig. 5, gray bars), which is not a significant difference compared to the control (100% residual PFU, Fig. 5, black and gray bars). In contrast, bacterial treatment with sodium periodate and the subsequent phage adsorption assay demonstrated a significant effect (*P* < 0.05) of sodium periodate on the ability of phages to adhere compared to the control sample with NaOAc (Fig. 6). Pretreatment of bacterial cells with 100 mM sodium periodate resulted in 87% (Fig. 6, black bars) and 92% (Fig. 6, gray bars) residual PFU (Fig. 6). Thus, only a small number of phages bound to pretreated bacterial cells, suggesting that LPS is necessary for phage binding. Corresponding to the lower concentration of 10 mM sodium periodate, percentages of 67% residual PFU (Fig. 6, black bars, phage IFTN4) and 86% residual PFU (Fig. 6, gray bars, phage IFTN3) were measured (Fig. 6). These effects were observed with significances of *P* < 0.025 and *P* < 0.0005 compared to the control sample NaOAc for both phages IFTN3 and IFTN4 (Fig. 6).

To further test whether the bacterial LPS is a receptor for *B. avium* phages, phages were treated with different amounts of LPS (Fig. S3). The results demonstrated that LPS isolated from the corresponding host strain prevented the infection with phages IFTN3 (Fig. S3A) and IFTN4 (Fig. S3B) in a dose-dependent manner. Cross-binding experiments showed that phage IFTN3 (Fig. S3C, gray) could also bind to LPS of the *B. avium* type strain CCUG 13726^T^ and phage IFTN4 (Fig. S3C, black) was able to bind to LPS of *B. avium* isolate 12/574/1/C, resulting in phage inactivation. These results confirmed our finding from the phage adsorption assay (Fig. 3), which showed equal adsorption of phages IFTN3 and IFTN4 to *B. avium* type strain CCUG 13726^T^ and *B. avium* isolate 12/574/1/C. Taken together, *B. avium* phages use LPS as a host receptor for phage adsorption.

### Determination of phage sizes by pulsed-field gel electrophoresis and family assignment.

Pulsed-field gel electrophoresis (PFGE) analysis was performed to estimate the size of the phage genomes and to determine whether bands indicative for linear DNA were visible. For all phages, a band was seen in the PFGE gel, and the approximate sizes of the bands ranged from 39 to 43 kb (Fig. S4). Circular DNA molecules of 30 kb or longer fail to enter pulsed-field gels (41, 42). Therefore, the genomes of all phages are linear double-stranded DNAs. In addition, genomic DNA of all seven phages was sequenced and revealed assembly sizes between 39,087 to 43,144 bp (Table 1), which were in the same range as the sizes estimated by PFGE analysis (Fig. S4). The GC content of phage DNAs was between 58.98% and 59.47%, while the GC content of *B. avium* 197N is slightly higher with 61.58%.

**FIG 7:**
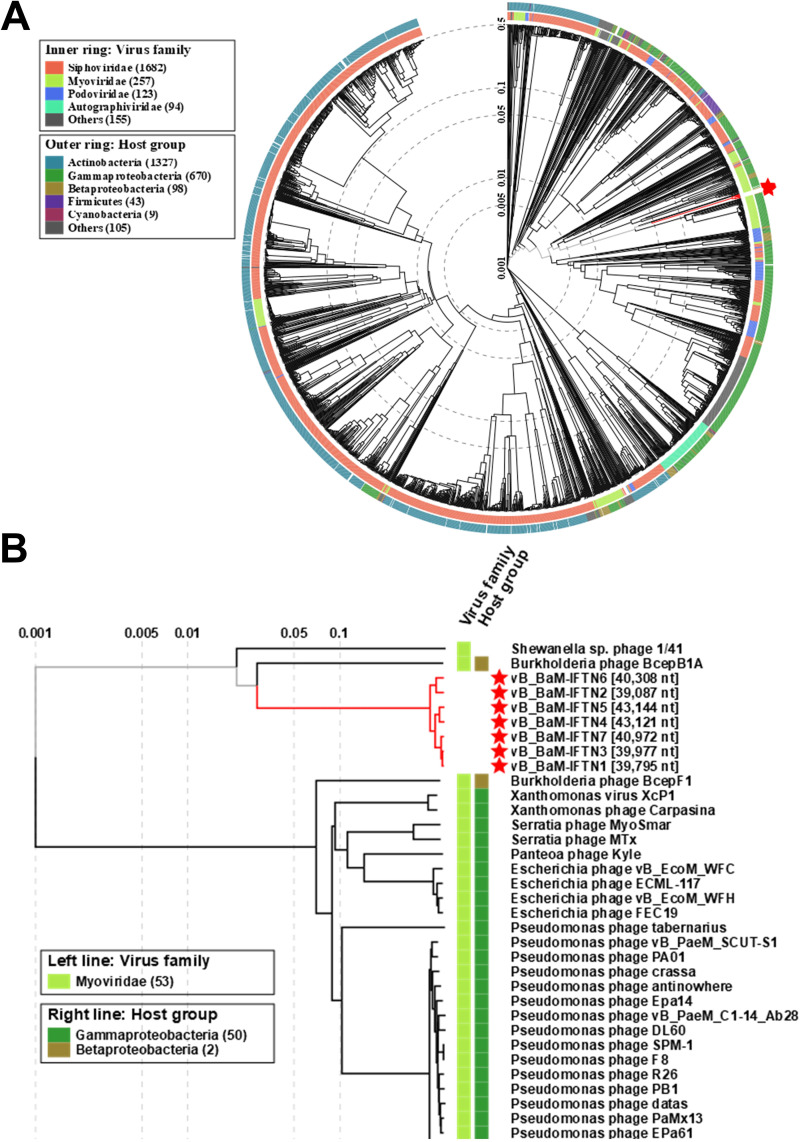
ViPTree proteomic tree of *B. avium* phages. (A) ViPTree circular proteomic tree of prokaryotic dsDNA viruses with *B. avium* phages. *B. avium* phage sequences are highlighted with red stars. (B) The rectangular presentation of the proteomic tree shows the closest related phages to vB_BaM-IFTN1-7 (IFTN1-7).

Comparison of phage genome sequences using VIRIDIC, a software which calculates pairwise intergenomic distances/similarities among viral genomes (43), revealed that all phages belong to the same genus (range of similarity from 71% to 97.8%) based on the phage genus demarcation criteria of 70% (44) (Fig. S5). To examine the evolutionary relationship of *B. avium* phages and the phages represented in the Virus-Host database (45), a viral proteomic tree based on full genome sequences was created using the Viral Proteomic Tree server (VipTree) (Fig. 7A). According to this analysis, all phages were located within the myoviruses, which was consistent with their morphological characteristics (Fig. 1). *Burkholderia* phage BcepB1A was evolutionary closely related to the *B. avium* phages (Fig. 7 B). However, comparison of the genome sequence of phage BcepB1A with genome sequences of phages isolated during the present study revealed a very low level (less than one percent) of similarity (Fig. S5).

### Comparison of the whole-genome sequences of *B. avium* phages.

**FIG 8:**
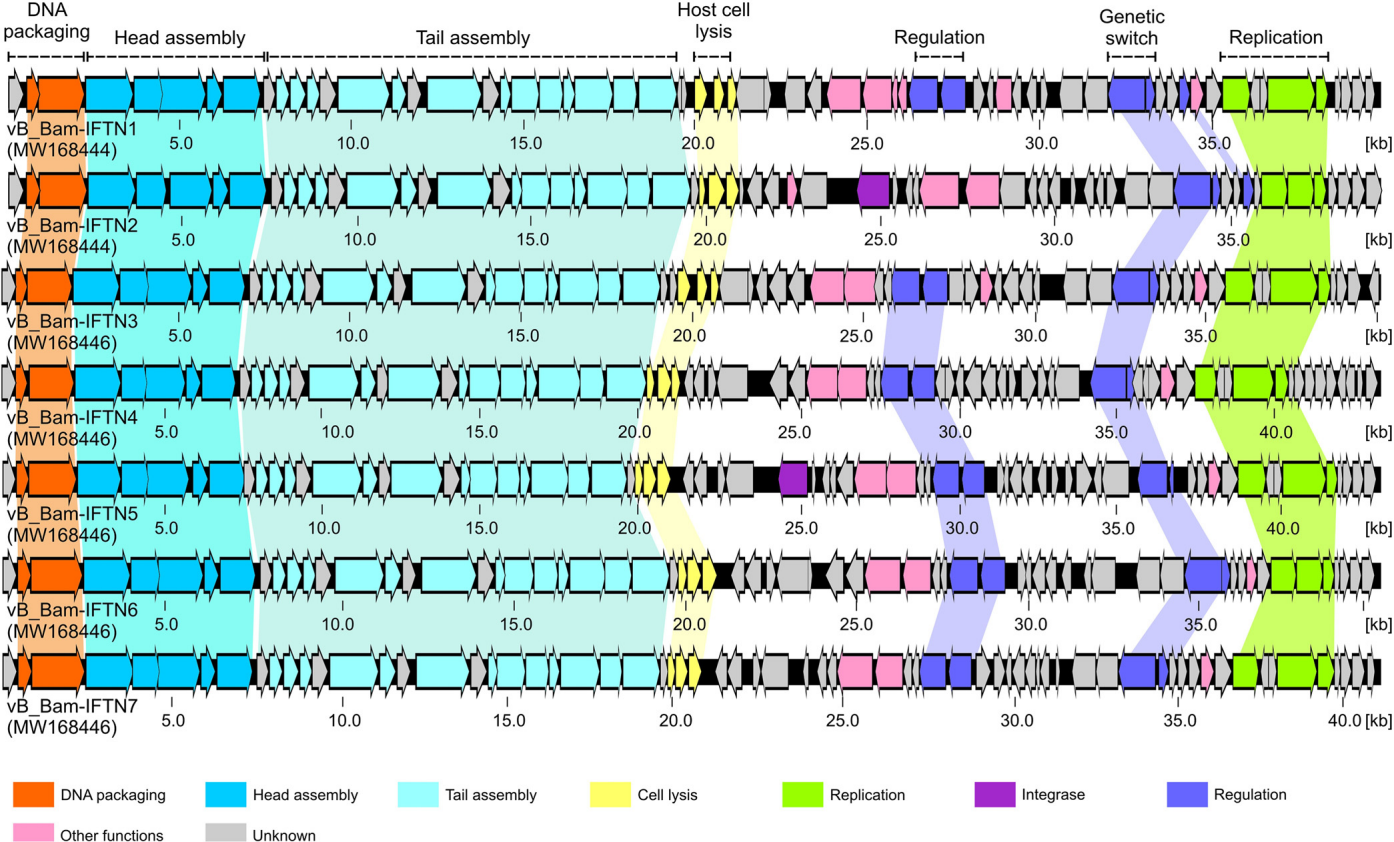
Genome organization of *B. avium* phages.

**FIG 9:**
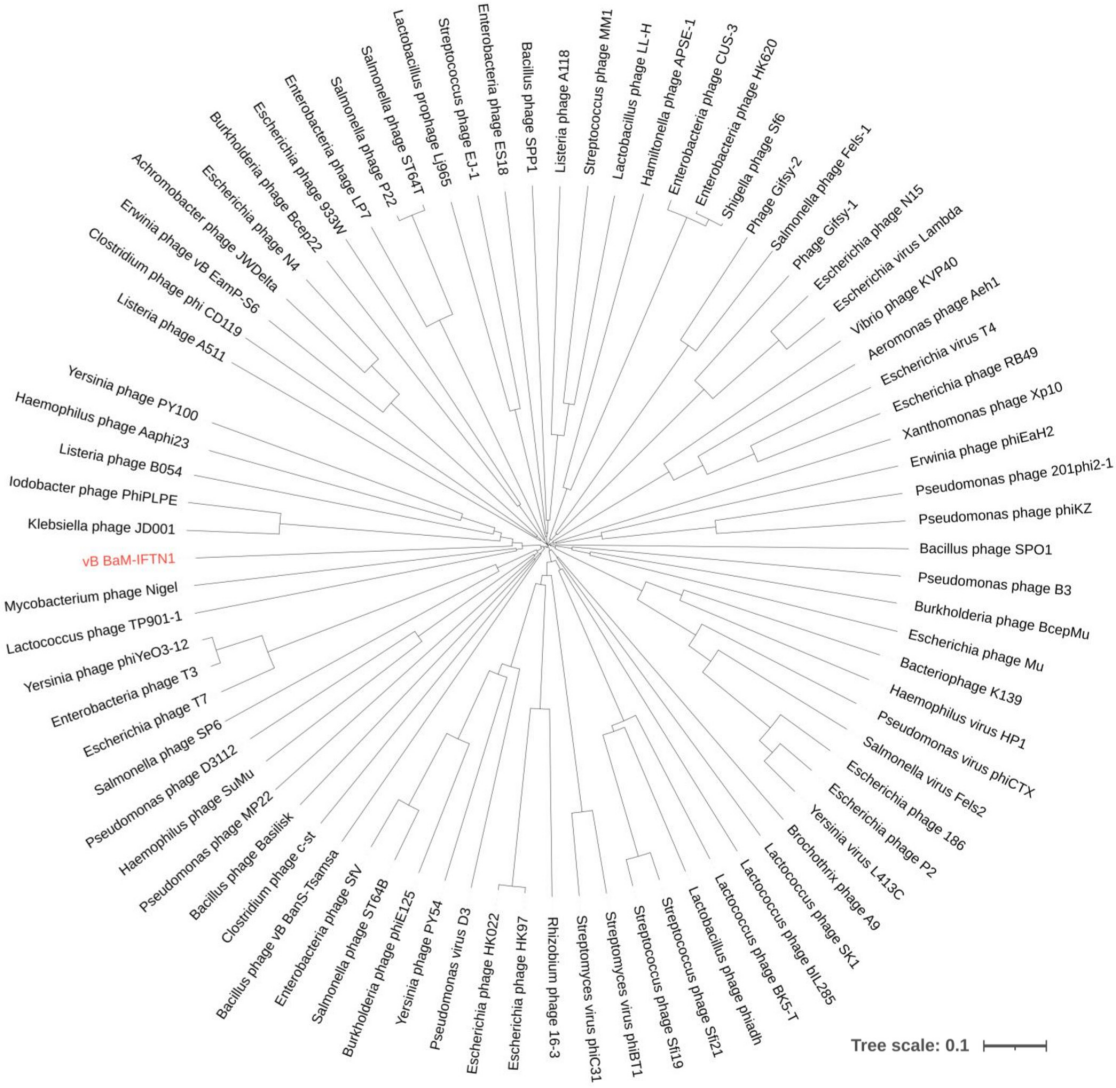
Phylogenetic tree of large terminase subunit (TerL) amino acid sequences. The sequences of 80 reference phages with the experimentally identified packaging strategy were selected based on previously published studies (46). The tree was created by the Simple Phylogeny tool based on the MUSCLE Multiple Sequence Alignment tool (90) and drawn using the Interactive Tree Of Life (iTOL) (91).

Analysis of genome sequences revealed that the phages are colinear with a conserved order of modules (Fig. 8). An overview of different phage modules is given in Table S1 in the supplemental material. For comparison of the DNA packaging module, a phylogenetic tree was built using TerL protein sequences of 80 reference phages with experimentally identified packaging strategies (46) together with TerL sequences of *B. avium* phages (Fig. 9). In this phylogenetic tree, *B. avium* phages clustered together with *Iodobacter* phage φPLPE (47), Klebsiella phage JD001 (48), *Listeria* phage B054 (49), Haemophilus phage AaΦ23 (50), and *Yersinia* phage PY100 (51). All these phages demonstrated a headful DNA-packaging strategy, and their genomes are terminally redundant and circularly permuted. Thus, our data indicated that the phages isolated in this study possess permuted genomes that are packaged according to the headful packaging strategy.

The region next to the DNA packaging genes encoded proteins for the head assembly (Fig. 8). The functions of two ORFs could not be determined from sequence comparisons (IFTN1-7_06 and IFTN1-7_07). However, HHpred analysis revealed that IFTN1-7_06 and IFTN1-7_07 might be related to the prohead core protein serine protease S77 (HHpred probability, 96.51%; E value, 0.018) and a capsid fiber protein of *Bacillus* phage phi29 (HHpred probability, 98.45%; E value, 1.5 × 10^−5^), respectively. The following sequence region encoded proteins for the tail and whole virion assembly (Fig. 8). ORFs IFTN1-7_12 and IFTN1-7_13 were similar to the head-tail adaptor gp16 (HHpred probability, 99.04%; E value, 3.3 × 10^−8^) and gp17 (HHpred probability, 96.21%; E value, 0.17) of *Bacillus* phage SPP1, respectively. ORFs IFTN1-7_14 and IFTN1-7_15 encoded most probably tail sheath (HHpred probability, 100%; E value, 2.1 × 10^−66^) and tube (HHpred probability, 99.42%; E value, 2.0 × 10^−11^) proteins, respectively, followed by a phage tail assembly chaperone protein (HHpred probability, 98.05%; E value, 6.0 × 10^−5^). Finally, seven ORFs from IFTN1-7_18 to IFTN1-7_24 encoded proteins for baseplate assembly. ORFs IFTN1-7_18, IFTN1-7_19, and from IFTN1-7_22 to IFTN1-7_24 showed sequence similarity to the baseplate organization protein gp11 (HHpred probability, 99.93%; E value, 1.7 × 10^−24^), baseplate stabilizing protein gp12 (HHpred probability, 99.95%; E value, 2.5 × 10^−26^), tail sheath initiator protein gp15 (HHpred probability, 99.93%; E value, 8.3 × 10^−24^), baseplate wedge protein gp16 (HHpred probability, 100%; E value, 3.8 × 10^−43^), and baseplate wedge protein, gp17 (HHpred probability, 100%; E value, 3.3 × 10^−36^) of *Vibrio* phage XM1 (GenBank accession number MT720689), respectively. ORFs IFTN1-7_20 and IFTN1-7_21 showed sequence identity to the protein of extracellular contractile injection system (HHpred probability, 99.93%; E value, 9.2 × 10^−23^) and to the puncturing protein gp41 of Pseudomonas phage SN (GenBank accession number NC_011756) (HHpred probability, 99.98%; E value, 2.6 × 10^−29^), respectively.

Despite the similarities between the DNA packaging modules, the head module, the tail module, the whole virion assembly module, and the base plate assembly module, there are differences in genes whose products encode proteins for host cell lysis and phage integration (Fig. 8). This module from phages IFTN1 and IFTN3 consist of four genes encoding holin (HHpred probability, 96.10%; E value, 0.02), SAR endolysin (endolysin containing signal-arrest-release signal [52]), and two spanins (53). However, comparison of this region to the corresponding regions of the other identified phages showed that it is shorter in the latter and containing only three genes encoding holin, SAR endolysin, and a spanin. Only two phages, IFTN2 and IFTN5, possessed genes encoding an integrase. The respective ORFs from phages IFTN2_35 and IFTN5_34 showed similarity to tyrosine integrases from different bacteria and phages and contain integrase-specific motifs (54). Sequence analysis did not reveal any genes for phage-like integrases in the other sequenced phages. Further sequence analyses revealed the presence of regions in the phage genomes that are associated with the regulation of life cycle switch and replication (Fig. 8). All phages harbored genes that might encode phage repressor proteins (IFTN1_49 in phage IFTN1) and Cro/CI family transcriptional regulators (IFTN1_50 in phage IFTN1). More interesting, all phages except IFTN2 contained two genes encoding antirepressor proteins (ORFs IFTN1_39 and IFTN1_40 in phage IFTN1).

### Lysogeny studies determining the life cycle of the identified phages.

Lysogeny studies were performed because there was an inconsistency between the whole-genome sequencing results and the phage phenotype determined in the spot assay. The analysis of whole-genome sequences revealed that all phages must be attributed to the temperate lifestyle. This is indicated by the presence of genes encoding integrases (phages IFTN2 and IFTN5) and antirepressor proteins (all phages except IFTN2) in the genomes of the phages. However, the phages IFTN1 to 7 demonstrated a clear plaque morphology in the spot assay with *B. avium* strain CCUG 13726^T^ and phages IFTN1 and IFTN3 to 6 also with *B. avium* isolate 12/574/1/C (Fig. 2). We could not observe any occurrence of turbidity inside the spots even after 48 h of incubation (55). For clarification, we streaked the bacteria from the lysis zone of IFTN4 onto fresh TSA agar plates. At least 20 bacterial colonies were selected after 24 h of incubation and examined for the presence of phage genome sequences. For this purpose, specific primers were used targeting the phage genome-encoded antirepressor protein. The results from the PCR assays showed that all colonies were negative for phage DNA. Therefore, another lysogeny assay according to Petrova and coworkers (2015) was applied (56). The frequency of lysogenization determined in this experiment varied from 2.3 to 84.3% depending on the phage used (Table 2). We tested 10 colonies from each assay for the presence of integrated phage DNA and for spontaneous phage release. All clones were positive for phage DNA and demonstrated spontaneous phage release (data not shown). Thus, selected phages may undergo a lysogenic life cycle with different frequency.

**TABLE 2:** Phage lysogenization rates

Phage	Lysogeny (%)
vB_BaM-IFTN1	7.1 ± 1.3
vB_BaM-IFTN2	57.0 ± 6.9
vB_BaM-IFTN3	84.3 ± 2.5^*a*^
vB_BaM-IFTN4	3.2 ± 2.1
vB_BaM-IFTN5	2.3 ± 0.8
vB_BaM-IFTN6	6.8 ± 1.5
vB_BaM-IFTN7	69.2 ± 15.2

a *B. avium* isolate 12/574/1/C was used for infection with vB_BaM-IFTN3 (IFTN3). *B. avium* strain CCUG 13726^T^ was used for infection with other phages.

Since it is known that the lysogenic state can be stable and persists after repeated passages (57), the stability of lysogeny was tested by both PCR for the presence of phage DNA and spontaneous phage release. We observed that the lysogenic state persisted after eight passages in the case of *B. avium* strain CCUG 13726^T^ and isolate 12/574/1/C infected with IFTN4 and IFTN3, respectively. To investigate the localization of phage DNA in the bacterial cells, we performed PFGE-based Southern blot analysis. Total DNA from lysogenic bacteria was prepared after passages 2, 4, and 6 and treated with the rare-cutting restriction endonuclease PmeI. This restriction endonuclease produces five fragments after digestion of *B. avium* chromosomal DNA and has no recognition site in the phage genomes. As a probe, we used a 331-bp internal fragment of the gene encoding the phage antirepressor (Ant) protein. The tested lysogenic bacteria possessed linear DNA of a size corresponding to the size of the phage genome, indicating active spontaneous release of phages, especially during the two first passages (Fig. S6). Furthermore, Southern blot analysis demonstrated that lysogenic bacteria contained phage DNA integrated into the chromosome (Fig. S6). In addition, we performed a spot assay to determine whether lysogenic *B. avium* isolates are resistant to the lysis by original phages. We were able to lyse the lysogenic bacteria regardless of the number of preceding passages, albeit with lower efficiency compared to using the parental nonlysogenic bacterial isolate. Passages of phages in lysogenic hosts significantly increased the efficiency of infection. Thus, integration of the phage genome into the bacterial chromosome does not result in immunity against superinfection.

## DISCUSSION

Infections with multidrug-resistant bacteria are a considerable threat for human and animal health. Therefore, the ongoing development of treatment alternatives such as phage therapy plays an important role in terms of the One Health concept (58). In this study, we describe the isolation and characterization of seven *B. avium* phages from the environment of poultry farms. Initial phenotypic analyses indicated that their broad host range and the lytic effect make these phages promising candidates for biocontrol of *B. avium.*

In general, seven different host range types have previously been described for phages, including adsorptive, penetrative, bactericidal, productive, plaquing, spotting, and lysogenic (59). In this study, we used spotting (or a spot assay) to determine the host ranges of the newly isolated phages. All phages were able to lyse several bacteria from the isolate collection (Fig. 2). Based on the determined ranges, the isolated phages could be divided into two groups. The first group, comprising all phages except IFTN3, was able to lyse a large set of *B. avium* field isolates and type strain CCUG 13726^T^. The second group consists of only one phage, IFTN3, which lysed a set of 13 field isolates (Fig. 2). Surprisingly, phages from both groups adsorbed to the tested bacteria with similar efficiency (Fig. 3). Therefore, the differences that we observed in the spot assay might be explained by the differences in their penetrative, bactericidal, or productive type of host range.

Morphological analysis was determined by transmission electron microscopy (Fig. 1) and suggested that all isolated *B. avium* phages are myoviruses, which was confirmed by a viral proteomic tree based on full genome sequences (Fig. 7). In one previous study by Shelton and colleagues (34), two temperate prophages, Ba1 and Ba2, were isolated, of which Ba1 was characterized in more detail. Similar to our findings, the morphology of Ba1 was found to be closest to members of the *Myoviridae* family and it was characterized as having an icosahedral head as well as a sheathed, contractile tail. Its genome size of 46.5 kb (34) was also close to, but slightly larger than the genome assembly sizes of the phages isolated in the present study, which ranged between 39 and 43 kb.

Comparison of genome sequences of the phages isolated in our study revealed a strong similarity in genes that encode the proteins for head and tail assembly (Fig. 8). This observation correlated with the similar morphology of all phages as determined by transmission electron microscopy and the comparable efficiency of phage adsorption to the host (Fig. 3). Moreover, a periodate treatment assay indicated that isolated phages bind to the same receptor and we found that isolated phages used the LPS as receptor (Fig. 5). LPSs are complexes that consist of three parts: lipid A, the O-polysaccharide, which is highly variable, and the core polysaccharide. Bacteria that contain all three components of the LPS are designated smooth (S) type and those that lack the O-polysaccharide portion belong to rough (R) type (60). Phages specific to S-type strains tend to target the O-polysaccharide and, due to the notable variability within this antigen, generally have a narrow host range (25). Regarding *B. avium* phages specifically, it was previously published that the LPS core and/or O-antigen could play a direct role in binding of *B. avium* phage Ba1 to the host bacteria (35). Even though *B. avium* cells do belong to the S type (61, 62), isolated phages nevertheless exhibited a broad host range in our study (Fig. 2), suggesting that the core polysaccharide, rather than the O-antigen, may be the receptor of *B. avium* phages.

Further genome analyses revealed the presence of genes for a temperate life cycle in all isolated phages: genes encoding an integrase, Cro/CI repressor, and antirepressors (63). Notably, the phages IFTN1, IFTN4, and IFTN6 did not harbor genes for integrases in their genomes, suggesting that an ORF with yet unknown function takes over this role. The detection of these genes highlights the importance of whole-genome sequencing and lysogeny studies independent of phenotype analyses to obtain clarity on the phage life cycle (64). Since no turbid plaques were visible in the spot assay, which might be indicative of a temperate life cycle, the question arises as to the differences between the lysogeny studies and the spot assay. The absence of a superinfection distinguishes our lysogenization experiment from the spot analysis. Superinfection occurs when another phage introduces its genetic material into lysogenic bacteria. To avoid superinfection, many phages have developed different mechanisms to protect bacteria from superinfection, which is termed superinfection exclusion (65, 66). Some phages produce proteins to mask cell surface receptors and block new infections. An example is phage T5, which forms lipoprotein Llp that inactivates its own receptor, the outer membrane protein FhuA (67). Other phages produce membrane-associated proteins to target and block the entry of phage DNA into the bacterial cell (68). Prophages produce repressor proteins that bind phage DNA and inhibit genes essential for phage replication (65). However, all lysogenic *B. avium* strains were susceptible to superinfection, although it remains to be shown that this also applies to nonpassaged *B. avium*. This finding opens a promising perspective for the therapeutic use of these phages. The therapeutic use of phages is based on criteria such as appropriate phage selection, isolation, and purification, killing efficacy of bacteria and their host range as well as their stability (69, 70). The property of lysogeny is generally undesirable (71, 72). For example, it was recently reported that the prophage content of Aeromonas salmonicida subsp. *salmonicida* can affect the efficacy of a cocktail of virulent phages for phage therapy (73). Analysis of 25 complete genome sequences of *B. avium* isolates available in the NCBI database (https://www.ncbi.nlm.nih.gov/genome/browse/#!/prokaryotes/1085/) for the presence of prophages using the PHASTER web server (74) revealed that most of these isolates did contain one or more prophages integrated into their chromosomal DNA (data not shown). Yet, despite this common feature, the isolated phages were able to kill all 51 *B. avium* isolates from our collection (Fig. 2). Ultimately, even though the *B. avium* phages isolated in this study can lysogenize the host, resulting lysogens do not develop a stable immunity against superinfection. This lack of superinfection immunity, which appears to be characteristic for *B. avium* phages, could then allow a new infection and bacterial lysis. Perhaps this should be included in the considerations as to whether only strict lytic phages can be used, and precise criteria should be defined in this regard. However, this also requires that the phages are genome-sequenced and do not carry virulence or antibiotic resistance genes, which needs to be monitored consequently. Overall, it can be concluded that the lack of development of superinfection immunity after lysogenization is a promising characteristic regarding the possibility of phage use in *B. avium* therapy. However, further research is needed to understand the mechanism underlying the lack of superinfection immunity after lysogenization and to determine whether *B. avium* phages are suitable for therapeutic purposes or if there are any limitations or challenges.

## MATERIALS AND METHODS

### Bacterial strains and culture conditions.

The *B. avium* type strain CCUG 13726^T^ (Culture Collection University of Gothenburg) and *B. avium* field isolates 16/29/1/B, 12/574/1/C, and X1131/2a were used for phage isolation. Another 47 *B. avium* isolates from a strain collection of the Institute for Veterinary Food Science were used for host range analysis. All 50 field isolates were isolated between 2002 and 2020 from different poultry species, originating from different geographical regions in Germany, Poland, and the Netherlands (13). The *B. avium* isolates were cultured on tryptic soy agar (TSA; Chemsolute, Renningen, Germany) or in tryptic soy broth (TSB; Chemsolute, Renningen, Germany) at 37°C.

### Phage isolation and purification.

Samples of drinking water (*n* = 8) and feces (*n* = 1) from seven noncommercial and two commercial chicken and turkey farms located in three regions of Germany were used to screen for phages capable of lysing *B. avium* strains. For this, the double agar overlay plaque assay was used as described previously (75, 76). To isolate phages, fecal samples were mixed with SM buffer (100 mM NaCl, 8 mM MgSO4, 50 mM Tris-HCl pH 7.5, 0.01% gelatin), incubated for 30 min at room temperature, and centrifuged for 30 min at 15,000 × *g*. Phages from drinking water and supernatants of fecal samples were precipitated with polyethylene glycol (PEG) and sodium chloride (NaCl) (stock solution of 20% PEG and 2.5 M NaCl) after filtration through a 0.45-μm filter. Aliquots (1 mL) of precipitates together with 1 mL of a *B. avium* CCUG 13726^T^ type strain culture (OD_600_ 1.0) were mixed with 5 mL of 0.6% TSB soft agar (TSB + 0.6% agar agar; 50°C) and poured onto dried TSA agar plates. The plates were incubated overnight at 37°C to form plaques (75, 76). Individual plaques were picked and eluted in SM buffer. The phage purification was repeated three times. The purified phages were propagated to concentrations ranging from 3 × 10^9^ to 3 × 10^10^ PFU mL^−1^ and stored at 4°C (77). For the following experiments, the phage titer was determined by the double agar overlay plaque assay method by adding 100 μL of a 10-fold dilution series of the phage and 0.5 mL bacterial suspension to 5 mL of 0.6% TSA soft agar. After the mixture was poured onto TSA agar plates, overnight incubation at 37°C was performed and plaques were subsequently counted.

### Electron microscopy.

Transmission electron microscopy (TEM) images of the phages were done at the Helmholtz Centre for Infection Research, Central Facility for Microscopy, Braunschweig, Germany. Thin carbon support films were prepared by evaporating a carbon thread onto a freshly cleaved mica surface (78). Small pieces of mica were then cut, and phages were negatively stained with 2% (wt/vol) aqueous uranyl acetate, pH 5.0. The samples were examined at an acceleration voltage of 80 kV in a TEM 910 transmission electron microscope (Carl Zeiss, Oberkochen, Germany). The phenotypic classification of phages was done using the morphological criteria of Ackermann (79, 80) and the International Committee of Taxonomy of Viruses (39, 40).

### Phage DNA extraction and whole-genome sequence analysis.

Phage DNA was extracted from high-titer stocks (approximately 3 × 10^10^ PFU mL^−1^) of phages using a phage DNA isolation kit (Norgen Biotek Corp., Thorold, ON, Canada) according to the manufacturer’s instructions. DNA sequencing was performed at the Federal Institute for Risk Assessment (BfR), Berlin, Germany using Next Generation Sequencing (Illumina) as previously described (81). DNA sequencing libraries were prepared using the Nextera Flex DNA sample preparation kit and subjected to short-read sequencing (2 × 151 cycles) on an NextSeq 500 (Illumina, San Diego, CA, USA). Raw read trimming and *de novo* genome assembly was conducted using the Aquamis pipeline. Prediction of coding sequences (CDS) was performed with the annotation tool of PATRIC (www.patricbrc.org).

### Host range determination.

The collection of 51 *B. avium* isolates, including the type strain was evaluated for susceptibility to isolated phages by spot testing on a double agar overlay, as described previously (82). Hence, 0.5 mL of target bacterial suspension (OD_600_ = 1.0) supplemented with 20 μL 100 mM MgSO_4_ and 20 μL 100 mM CaCl_2_ were mixed with 5 mL of 0.6% TSA soft agar, poured onto a TSA agar plate, and allowed to solidify. Then, 10 μL of each phage lysate (in serial 10-fold dilutions of the stock suspensions) was dropped onto the overlay, dried, and cultured at 37°C for 20 h. The host range of the phages was determined based on the presence or absence of plaques.

### Phage adsorption.

To study the kinetics of phage adsorption, bacteria were resuspended in TSB (adjusted to 5 × 10^8^ CFU mL^−1^), before 50 μL of phage lysate (MOI = 1.0) was added. After an adsorption time of 20 min at 37°C, samples were centrifuged (17,000 × *g* for 1 min) to sediment the bacterial cells. A volume of 100 μL of the diluted supernatant was assayed for nonadsorbed phages using the double agar overlay plaque assay method. The plaques formed were counted and compared to the control sample without bacterial cells. Each assay was performed three times independently.

### Phage bacteriolytic activity.

Bacterial growth curves based on spectrophotometric measurements of the optical density at 600 nm (OD_600_) were generated to determine the bacteriolytic activity of the phages (77). Phages were added to bacterial suspensions (initial OD_600_ of 0.1) with an MOI of 1 and 0.1 in TSB medium and bacterial suspensions without phage infection ran along as negative controls. Samples were incubated for 24 h at 37°C in a shaking incubator. OD_600_ measurements were performed hourly for a period of 8 h and one was taken after 24 h. The samples were incubated at 37°C in a shaking incubator. The experiment was performed three times independently. In addition, growth curves were compiled based on measurements of bacterial colony counts. Measurements were taken hourly over a period of 8 h, with further measurements taken after 24 and 48 h. This experiment was performed in duplicate.

### Bacterial treatment with proteinase K or sodium periodate.

To determine the structure of the phage receptor, bacteria were treated with proteinase K or sodium periodate, followed by phage adherence experiments, as described earlier (83). To determine whether bacterial treatment with proteinase K affects phage adherence, 0.5 mL bacterial suspension (OD_600_ 1.0) was treated with proteinase K (0.2 mg mL^−1^; Qiagen, Hilden, Germany) for 3 h at 37°C. After centrifugation (1 min, 17,000 × *g*), the pellet was washed with TSB medium and resuspended in 0.5 mL TSB medium. The bacteria were then infected with phages at an MOI of 1 for 1 h at 37°C. The number of phages remaining in the supernatant was measured after centrifugation (1 min at 17,000 × *g*) using the double agar overlay plaque assay method. An untreated sample was included as a control. The experiment was repeated in three independent experiments in triplicate giving a total of nine replicates per group.

The influence of sodium periodate on phage adherence was tested by a method described previously (83). Hence, 0.5 mL bacterial suspensions (OD_600_ = 1.0) were centrifuged (1 min, 17,000 × *g*) and the pellets were resuspended in either 1 mL sodium acetate (NaOAc) (50 mM, pH 5.2) (Merck, Darmstadt, Germany), 1 mL of 10 mM sodium periodate (Sigma-Aldrich, Darmstadt, Germany), or 1 mL of 100 mM sodium periodate. Sodium periodate was dissolved in sodium acetate (NaOAc), thus, sodium acetate served as the control substance. The bacterial treatment was carried out for 2 h in the dark at room temperature (20°C). After centrifugation (1 min at 17,000 × *g*), pellets were washed once with TSB medium and resuspended in 0.5 mL TSB medium. Phage infection was carried out in the same way as after proteinase K treatment. One sample without sodium periodate and one with sodium acetate treatment was included as an experimental control. The experiment was repeated in three independent experiments in triplicate giving a total of nine repeats per group.

### Lipopolysaccharide extraction from bacterial cells.

Lipopolysaccharides (LPS) from *B. avium* cells were isolated by the Tri-Reagent method (84). For this, 10 mg of bacterial pellets were mixed with 0.2 mL QIAzol lysis reagent (Qiagen) according to the manufacturer’s instructions and the mixture was incubated at room temperature (RT) (10 to 15 min). Then, 20 μL chloroform per milligram bacterial pellet was added for phase separation. Strong vortexing and further incubation (10 mL, RT) was performed. The mixture was centrifuged (12,000 × *g* for 10 min) to separate the water and organic phases. The water phase was transferred to a new centrifuge tube. A volume of 100 μL distilled water was added to the organic phase, vortexed and incubated for another 10 min at RT. After centrifugation, the upper aqueous phases from both steps were combined. The combined aqueous phases were used for further experiments.

### Phage inactivation by LPS.

The binding ability of phages to LPS was tested by adding different amounts of LPS to phages and performing the double agar overlay plaque assay method. Briefly, 100 μL phage lysate (titer approximately 3 × 10^5^ PFU mL^−1^) was mixed with different amounts of prewarmed (37°C, 15 min) LPS and incubated for 1 h at 37°C. In the following, 0.5 mL bacterial suspension (ca. 5 × 10^8^ CFU mL^−1^) was infected with 10 μL of each phage-LPS mix and the double agar overlay plaque assay method was performed. Water instead of LPS was added to the control sample. This test was repeated three times independently.

### Lysogeny assays.

Overnight cultures of *B. avium* type strain CCUG 13726^T^ and isolate 12/574/1/C were diluted to an OD_600_ of 1.0 in TSB medium. Then, 10-fold serial dilutions were prepared until there were only 10^3^ to 10^4^ cells per milliliter, and 100 μL of each dilution step was plated onto TSA agar plates seeded with 10^9^ PFU mL^−1^ of phages IFTN4 and IFTN3, respectively, and a plate without phages. Plates were incubated at 37°C for 24 h and colonies counted. The ratio of colonies on phage-containing plates relative to plates without phages was used to determine lysogeny frequencies. To test isolates for spontaneous phage release, individual colonies were streaked onto a top agar layer containing the corresponding *B. avium* isolate. Negative-control plates were prepared for each putative lysogen by streaking the bacterial samples on plates without bacteria to ensure the growth of the streaked bacteria.

Lysogeny PCR was performed to identify whether the isolated lysogenic bacteria harbored the phage genome. A master mix was prepared, including the forward primer 5′-TGT ATT AAG TTG TCG CTC GCA CTC -3′ and the reverse primer 5′-GCC TCA TCA CTA TCA ACC AAG CC -3′ that amplified a fragment of the phage gene encoding an antirepressor protein (ORF 39 in IFTN1). PCR conditions involved a preheating step at 95°C for 5 min, 30 cycles at 95°C for 30 s, 61°C for 30 s, and 72°C for 30 s. DNA from uninfected bacteria was used as a negative control. All amplicons were run on a 1.5% agarose gel.

### PFGE and Southern blot analysis.

Macrorestriction analysis of *B. avium* genomes digested with the restriction nucleases PmeI (New England Biolabs Inc., Hitchin, United Kingdom) was performed based on a previously published protocol (13, 85). Digested DNA from Salmonella Typhimurium LT2 was used as a marker (86). The fragments were separated for 20 h in a CHEF DR II system (Bio-Rad, Hercules, United States) at 6 V, starting at an initial time of 6.8 s and ending at a final time of 35.2 s. The DNA was transferred from PFGE gels to positively charged nylon membranes (Amersham Hybond-N+, GE Healthcare, Chicago, United States) by overnight capillary transfer with 20 × SSC (3.0 M sodium chloride, 0.3 M sodium citrate, pH 7.0) buffer. Probe labeling, hybridization, and Southern blot development was performed using the DIG high prime DNA labeling and detection starter kit I (for color detection with NBT/BCIP) (Roche Diagnostics, Mannheim, Deutschland) according to the manufacturer’s protocol. A 331-bp fragment of the gene encoding the antirepressor protein (ORF 39 in IFTN1) was amplified by using the primers chosen for the lysogeny PCR and was applied as a probe for Southern blot analysis.

### Bioinformatic and statistical analysis.

Phage homology calculations were performed using VIRIDIC software (http://rhea.icbm.uni-oldenburg.de/VIRIDIC/) (43). All phages were used to build a whole-genome tree with ViPTree software (version 3.0) and analyze their similarities. The ViPTree server (https://www.genome.jp/viptree) generates a “proteomic tree” of viral genome sequences based on genome-wide sequence similarities computed by tBLASTx (87). Additional annotation of the phage proteins was performed using the HHpred bioinformatics toolkit (https://toolkit.tuebingen.mpg.de/tools/hhpred) (88, 89).

The mean values and the standard deviations from the experimental results were calculated using Microsoft Excel 2016. The statistical significance of differences in results was calculated using the Student´s *t* test, where *P* values of <0.05 were defined as statistically significant.

### Data availability.

The nucleotide sequences of the phages were deposited in the GenBank database under the accession numbers OM293950 (IFTN1), OM293951 (IFTN2), OM293952 (IFTN3), OM293953 (IFTN4), OM293954 (IFTN5), OM293955 (IFTN6), and OM293956 (IFTN7).
