# Supplementary material for: Characterization of Temperate LPS-Binding Bordetella avium Phages That Lack Superinfection Immunity
Source: Microbiol Spectr. 2023 May 1;11(3):e03702-22. doi: 10.1128/spectrum.03702-22 (PMC10269795; doi:10.1128/spectrum.03702-22)
Supplement: Supplemental file 1 — Table S1 and Fig. S1 to S6. Download spectrum.03702-22-s0001.pdf, PDF file, 1.9 MB [file spectrum.03702-22-s0001.pdf]

SUPPLEMENTAL MATERIAL

Table S1. Comparison of whole genome sequences of *B. avium* phages.

Predicted function of gene products	Involved ORFs	Highest similarity of the deduced amino acid sequence to a	HHpred probability	Phages	Supplemental notes
DNA packaging	IFTN1-7_02<br>IFTN1-7_03	small terminase subunit<br>large terminase subunit		IFTN1 to IFTN7	References 46-51
head assembly	IFTN1-7_06<br>IFTN1-7_07	prohead core protein serine protease S77 of *Bacillus* phage phi29<br>capsid fiber protein of *Bacillus* phage phi29	96.51%; E value, 0.018<br>98.45%; E value, 1.5×10^{-5}	IFTN1 to IFTN7	
tail and whole virion assembly	IFTN1-7_12<br>IFTN1-7_13<br>IFTN1-7_14<br>IFTN1-7_15<br>IFTN1-7_16	head-tail adapter gp16 of *Bacillus* phage SPP1<br>head-tail adapter gp17 of *Bacillus* phage SPP1<br>tail sheath proteins<br>tube proteins<br>phage tail assembly chaperone protein	99.04%; E value, 3.3×10^{-8}<br>96.21%; E value, 0.17<br>100%; E value, 2.1×10^{-66}<br>99.42%; E value, 2.0×10^{-11}<br>98.05%; E value, 6.0×10^{-5}	IFTN1 to IFTN7	
baseplate assembly	IFTN1-7_18<br>IFTN1-7_19<br>IFTN1-7_22<br>IFTN1-7_23<br>IFTN1-7_24<br>IFTN1-7_20<br>IFTN1-7_21	baseplate organization protein gp11 of *Vibrio* phage XM1 [*)]<br>baseplate organization protein gp12 of *Vibrio* phage XM1 [*)]<br>tail sheath initiator protein gp15 of *Vibrio* phage XM1 [*)]<br>baseplate wedge protein gp16 of *Vibrio* phage XM1 [*)]<br>baseplate wedge protein gp17 of *Vibrio* phage XM1 [*)]<br>protein of extracellular contractile injection system of *Pseudomonas* phage SN [**)]<br>puncturing protein gp41 of *Pseudomonas* phage SN [**)]	99.93%; E value, 1.70×10^{-24}<br>99.95%; E value, 2.50×10^{-26}<br>99.93%; E value, 8.3×10^{-24}<br>100%; E value, 3.8×10^{-43}<br>100%; E value, 3.3×10^{-36}<br>99.93%; E value, 9.2×10^{-23}<br>99.98%; E value, 2.6×10^{-29}	IFTN1 to IFTN7	[*)] *Vibrio* phage XM1 (accession number MT720689)<br>[**)] *Pseudomonas* phage SN (accession number NC_011756)

FIG S1

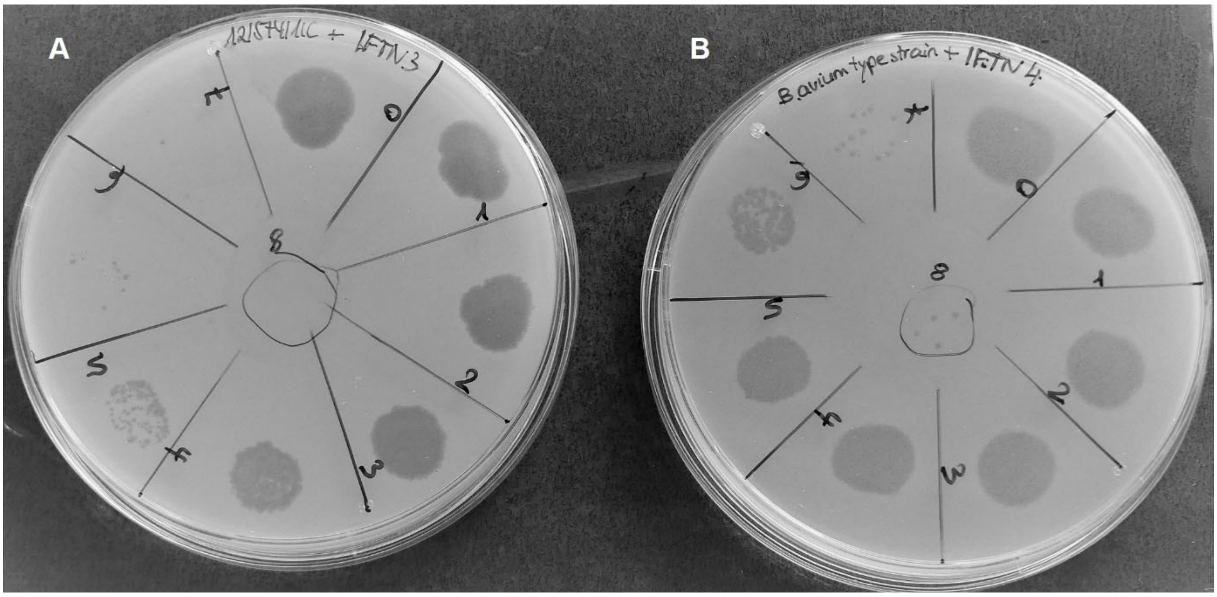

Spot assay using dilutions of phage stocks. *B. avium* isolate 12/574/1/C (A) and *B. avium* type strain CCUG 13726^T (B) were infected with phages vB_BaM-IFTN3 (IFTN3) and vB_BaM-IFTN4 (IFTN4), respectively. Phage suspensions were spotted onto TSA agar plates in serial tenfold dilution steps (indicated in the figures as 0 for the highest and 8 for the lowest phage concentration).

FIG S2

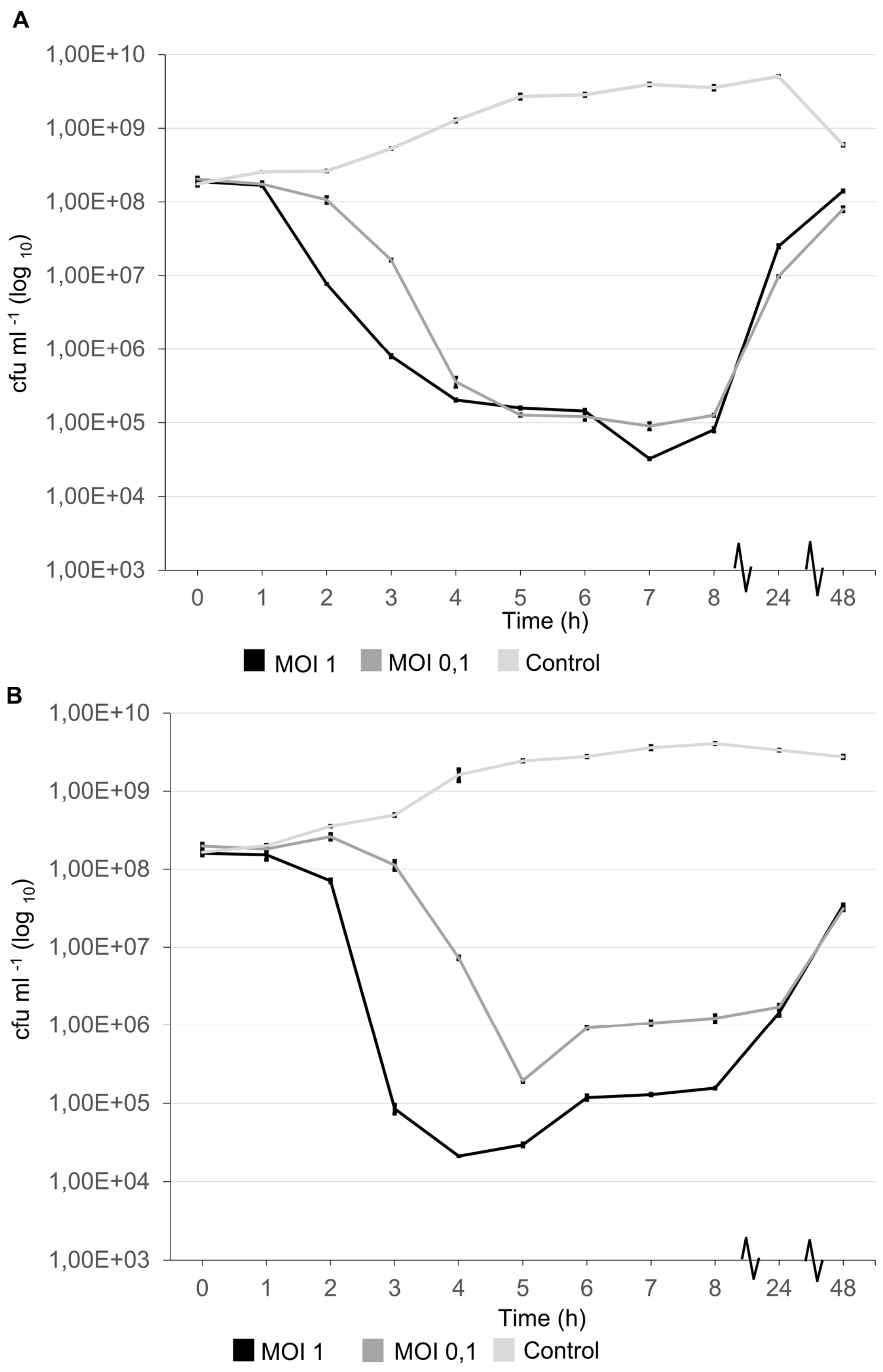

Bacteriolytic activity. The bacteriolytic activity of phage vB_BaM-IFTN4 (IFTN4) against *B. avium* type strain CCUG 13726^T (A) and phage vB_BaM-IFTN3 (IFTN3) against *B. avium* 12/574/1/C (B) are shown at MOI 1 and MOI 0.1, in reference to the bacterial growth curve. This experiment was set in duplicate (mean ± SD).

FIG S3

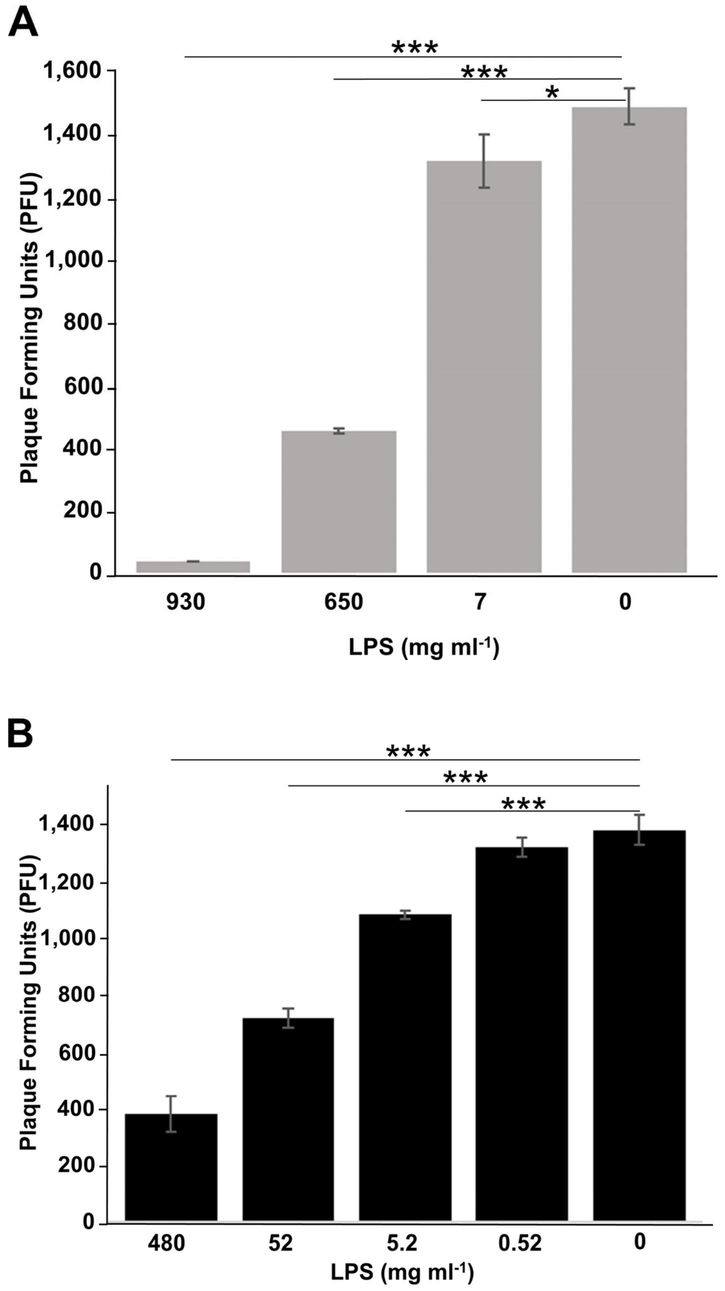
A
***
***
*
1,600
1,400
1,200
1,000
800
600
400
200
0
Plaque Forming Units (PFU)
930
650
7
0
LPS (mg ml-1)
B
***
***
***
1,400
1,200
1,000
800
600
400
200
0
Plaque Forming Units (PFU)
480
52
5.2
0.52
0
LPS (mg ml-1)

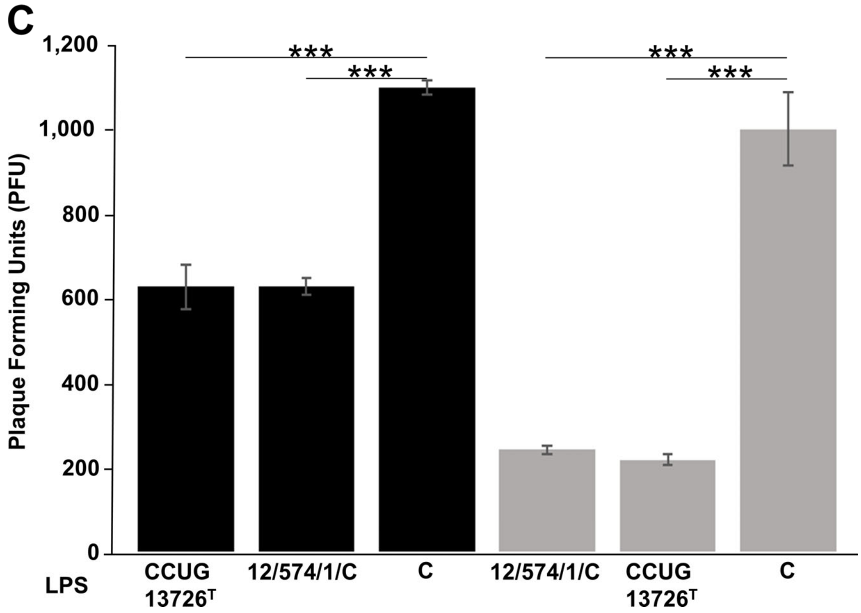

Phage inactivation by bacterial lipopolysaccharide (LPS). (A) Phage vB_BaM-IFTN3 (IFTN3) was incubated with different amounts of LPS from *B. avium* isolate 12/574/1/C. (B) Phage vB_BaM-IFTN4 (IFTN4) was incubated with different amounts of LPS from *B. avium* type strain CCUG 13726^T. (C) Phages vB_BaM-IFTN3 (IFTN3, grey panel) and vB_BaM-IFTN4 (IFTN4, black panel) were incubated with LPS of *B. avium* isolate 12/574/1/C and type strain CCUG 13726^T. C Control. After incubation, the double agar overlay plaque assays using phages vB_BaM-IFTN3 (IFTN3) and vB_BaM-IFTN4 (IFTN4) and *B. avium* isolate 12/574/1/C and type strain CCUG 13726^T, respectively, were performed. The scale bar is shown as amount of PFU. * $P < 0.05$, *** $P < 0.0005$. The results are the average of three independent experiments (mean ± SD).

FIG S4

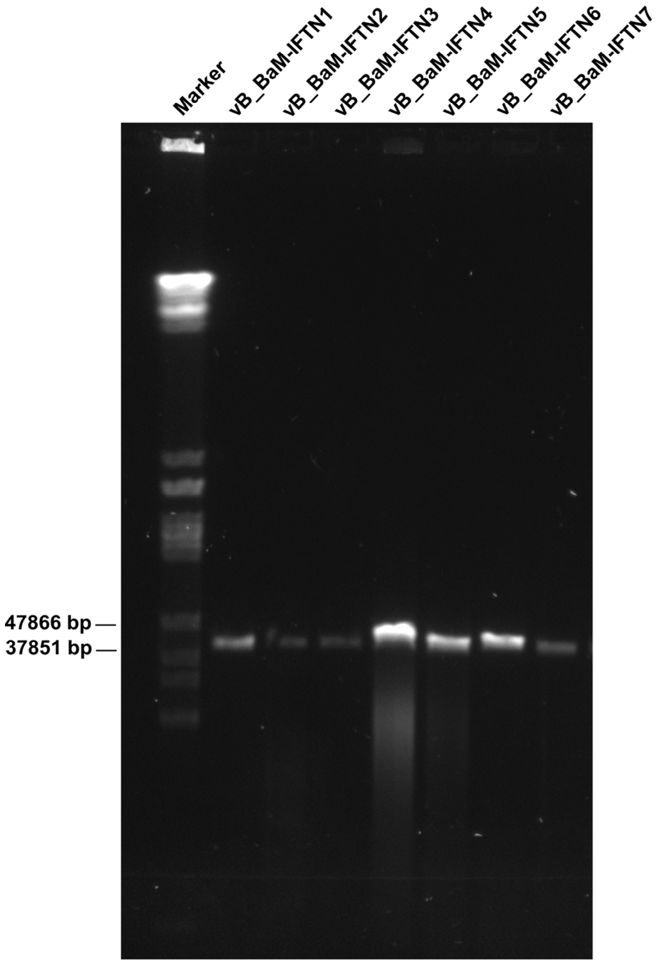

Pulsed-field gel electrophoresis (PFGE) of *B. avium* phage DNA. Chromosomal DNA of *Salmonella enterica* strain LT2 digested with restriction endonuclease XbaI was used as a marker.

FIG S5

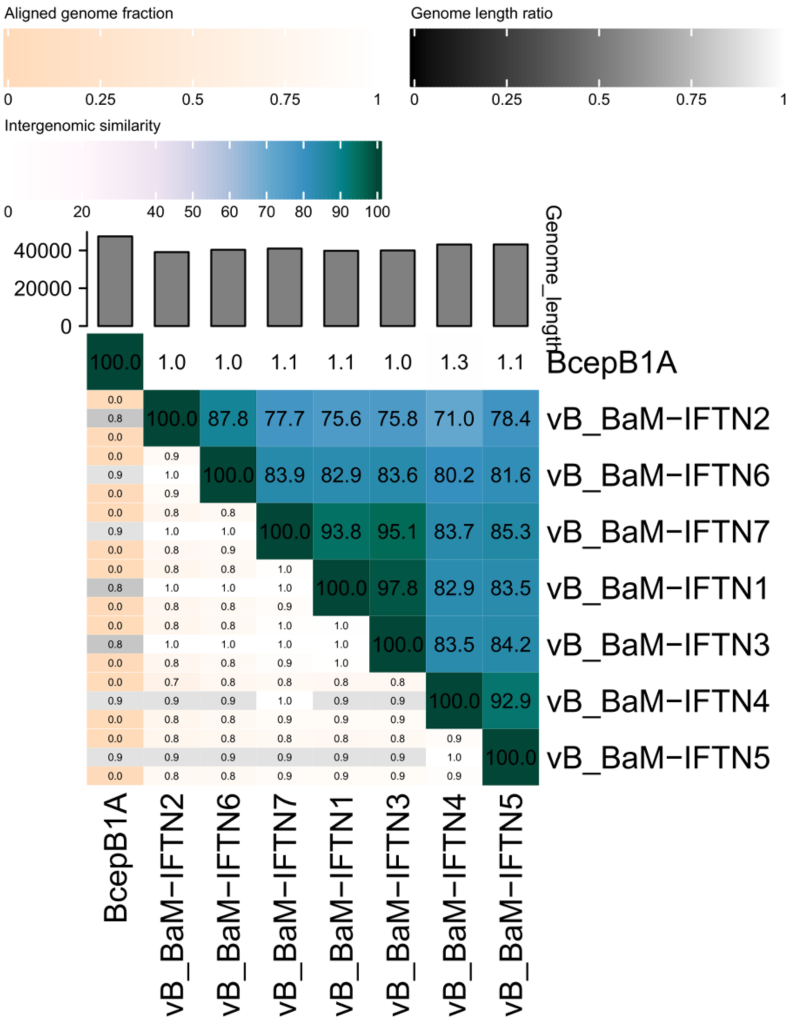

The whole-genome comparison of *B. avium* phages. The comparison and clustering were performed using Viridic (Virus Intergenomic Distance Calculator). Different shades of blue in the right half of the heatmap represent different intergenomic similarities (in %) between the genomes of each pair compared, as indicated above the heatmap and specified by numbers. The left half of the heatmap shows three indicator values for each genome pair: aligned fraction of genome 1 for the genome in this row (top value), genome length ratio for the two genomes in this pair (middle value) and aligned fraction of genome 2 for the genome in this column (bottom value). The darker colors represent lower values as indicated above the heatmap. BcepB1A - *Burkholderia* phage BcepB1A (GenBank accession number NC_005886).

FIG S6

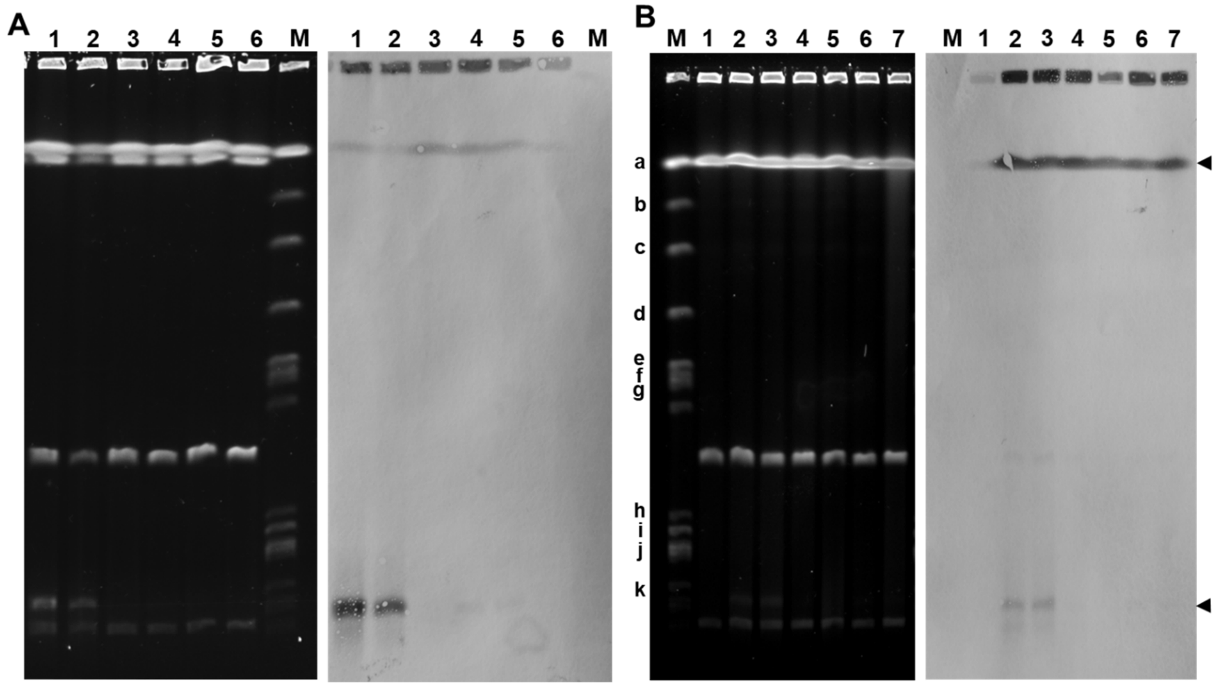

PFGE of total DNA from lysogenic *B. avium* strains digested with restriction endonuclease PmeI. *B. avium* strain CCUG 13726^T (A) and isolate 12/574/1/C (B) were infected with phages vB_BaM-IFTN4 (IFTN4) and vB_BaM-IFTN3 (IFTN3), respectively. Lysogenic clones were isolated, and total DNA was prepared after passages 2, 4, and 6 and digested with restriction endonuclease PmeI. A 331 bp fragment of the gene encoding the anti-repressor protein was applied as a probe for Southern blot analysis. (A) **lanes 1, 2** - passage 2; **lanes 3, 4** – passage 4**; lanes 5, 6** – passage 6; **M -** chromosomal DNA of *Salmonella enterica* strain LT2 digested with restriction endonuclease XbaI. (B) **M -** chromosomal DNA of *Salmonella enterica* strain LT2 digested with restriction endonuclease XbaI. **Lane 1** - *B. avium* isolate 12/574/1/C; **lanes 2, 3** - passage 2; **lanes 4, 5** - passage 4**; lanes 6, 7** - passage 6. XbaI fragments of *Salmonella enterica* strain LT2 (kb): **a** – 800, 708, 675; **b** – 457; **c** – 365; **d** – 275; **e** – 243; **f** -233; **g** – 225, 224; **h** – 104; **i** – 90; **j** – 72, 70; **k** – 49, 48 (86).
